# Glutamine Metabolism Supports the Functional Activity of Immune Cells against Aspergillus fumigatus

**DOI:** 10.1128/spectrum.02256-22

**Published:** 2022-12-08

**Authors:** Daniela Antunes, Samuel M. Gonçalves, Vasiliki Matzaraki, Cláudia S. Rodrigues, Relber A. Gonçales, Joana Rocha, Jorge Sáiz, António Marques, Egídio Torrado, Ricardo Silvestre, Fernando Rodrigues, Frank L. van de Veerdonk, Coral Barbas, Mihai G. Netea, Vinod Kumar, Cristina Cunha, Agostinho Carvalho

**Affiliations:** a Life and Health Sciences Research Institute (ICVS), School of Medicine, University of Minho, Braga, Portugal; b ICVS/3B’s—PT Government Associate Laboratory, Guimarães/Braga, Portugal; c Department of Internal Medicine and Radboud Center for Infectious Diseases, Radboud University Medical Center, Nijmegen, The Netherlands; d Centro de Metabolómica y Bioanálisis (CEMBIO), Facultad de Farmacia, Universidad San Pablo CEU, CEU Universities, Madrid, Spain; e Serviço de Imuno-Hemoterapia, Hospital de Braga, Braga, Portugal; f Department for Immunology and Metabolism, Life and Medical Sciences Institute (LIMES), University of Bonn, Bonn, Germany; g Department of Genetics, University of Groningen, University Medical Center Groningen, Groningen, The Netherlands; Universidade de Sao Paulo

**Keywords:** immunometabolism, glutamine, *Aspergillus*, macrophage, antifungal immunity

## Abstract

The reprogramming of cellular metabolism of immune cells is an essential process in the regulation of antifungal immune responses. In particular, glucose metabolism has been shown to be required for protective immunity against infection with Aspergillus fumigatus. However, given the intricate cross talk between multiple metabolic networks and signals, it is likely that cellular metabolic pathways other than glycolysis are also relevant during fungal infection. In this study, we demonstrate that glutamine metabolism is required for the activation of macrophage effector functions against A. fumigatus. Glutamine metabolism was found to be upregulated early after fungal infection and glutamine depletion or the pharmacological inhibition of enzymes involved in its metabolism impaired phagocytosis and the production of both proinflammatory and T-cell-derived cytokines. In an *in vivo* model, inhibition of glutaminase increased susceptibility to experimental aspergillosis, as revealed by the increased fungal burden and inflammatory pathology, and the defective cytokine production in the lungs. Moreover, genetic variants in glutamine metabolism genes were found to regulate cytokine production in response to A. fumigatus stimulation. Taken together, our results demonstrate that glutamine metabolism represents an important component of the immunometabolic response of macrophages against A. fumigatus both *in vitro* and *in vivo*.

**IMPORTANCE** The fungal pathogen Aspergillus fumigatus can cause severe and life-threatening forms of infection in immunocompromised patients. The reprogramming of cellular metabolism is essential for innate immune cells to mount effective antifungal responses. In this study, we report the pivotal contribution of glutaminolysis to the host defense against A. fumigatus. Glutamine metabolism was essential both *in vitro* as well as in *in vivo* models of infection, and genetic variants in human glutamine metabolism genes regulated cytokine production in response to fungal stimulation. This work highlights the relevance of glutaminolysis to the pathogenesis of aspergillosis and supports a role for interindividual genetic variation influencing glutamine metabolism in susceptibility to infection.

## INTRODUCTION

Invasive pulmonary aspergillosis (IPA) is a life-threatening fungal disease, mainly caused by the opportunistic fungal pathogen Aspergillus fumigatus (1). The development of IPA occurs typically as the result of severe immune dysfunction, including prolonged neutropenia or neutrophil functional impairment, prolonged corticosteroid therapy, and stem-cell or solid organ transplantation (2, 3). Recent studies have also revealed an increased frequency of invasive aspergillosis among critically ill patients with viral pneumonia, e.g., influenza and COVID-19 (4, 5). Diagnosis of IPA remains challenging and may compromise the timely administration of antifungal therapy (6), contributing to the high mortality rates of infected patients, estimated to be above 30% (7). Considering the increasing numbers of immunocompromised patients and the associated incidence of IPA, efforts aimed at improving the knowledge of the pathogenetic mechanisms that underlie this severe infection are required to expand our current possibilities for clinical intervention (8).

The reprogramming of the cellular metabolism is a crucial mechanism required by immune cells to counter infection (9, 10). In response to fungal infection, the functional activity of macrophages was recently demonstrated to depend on a metabolic shift toward glycolysis (11, 12). This rewiring of the cellular metabolism generates a rapid energy source required to activate specific antimicrobial programs, including the production of cytokines and the oxidative burst. However, oxidative phosphorylation remains often operational (13), supporting the possible involvement of other metabolic pathways in the immune response to fungal infection. Likewise, the stimulation of monocytes with β-1,3-glucan from the fungal cell wall and the induction of innate immune memory were found to implicate not only glycolysis (14) but other metabolic pathways such as glutaminolysis (15) and cholesterol metabolism (16).

Glutamine, the most abundant free amino acid in the human body, is a major source of energy for cells (17). The glutaminase enzyme converts glutamine into glutamate, which in turn can be converted to α-ketoglutarate by the glutamate dehydrogenase to feed the tricarboxylic acid (TCA) cycle. Glutaminolysis has been demonstrated to play an important role in the immune response to several pathogens. For example, the effector functions of macrophages in response to infection with HIV, Mycobacterium tuberculosis, and Leishmania donovani were found to rely to a large extent on glutamine metabolism (18–20). Importantly, innate immune responses to Candida albicans yeast, but not hyphae, were also found to require the induction of glutaminolysis (21).

In this study, we sought to investigate the relevance of glutamine metabolism to the host response against infection with the opportunistic fungal pathogen Aspergillus fumigatus. We demonstrate that glutaminolysis is an essential metabolic process required for the activation of antifungal effector functions of macrophages, namely, cytokine production and phagocytosis. Moreover, human genetic variants in glutamine metabolism genes were found to influence cytokine production in response to fungal stimulation. Collectively, our results reveal glutamine metabolism as an essential pathway in antifungal immunity and highlight it as a potential therapeutic target to prevent or treat IPA.

## RESULTS

### Glutamine metabolism is upregulated in A. fumigatus-infected macrophages.

**FIG 1:**
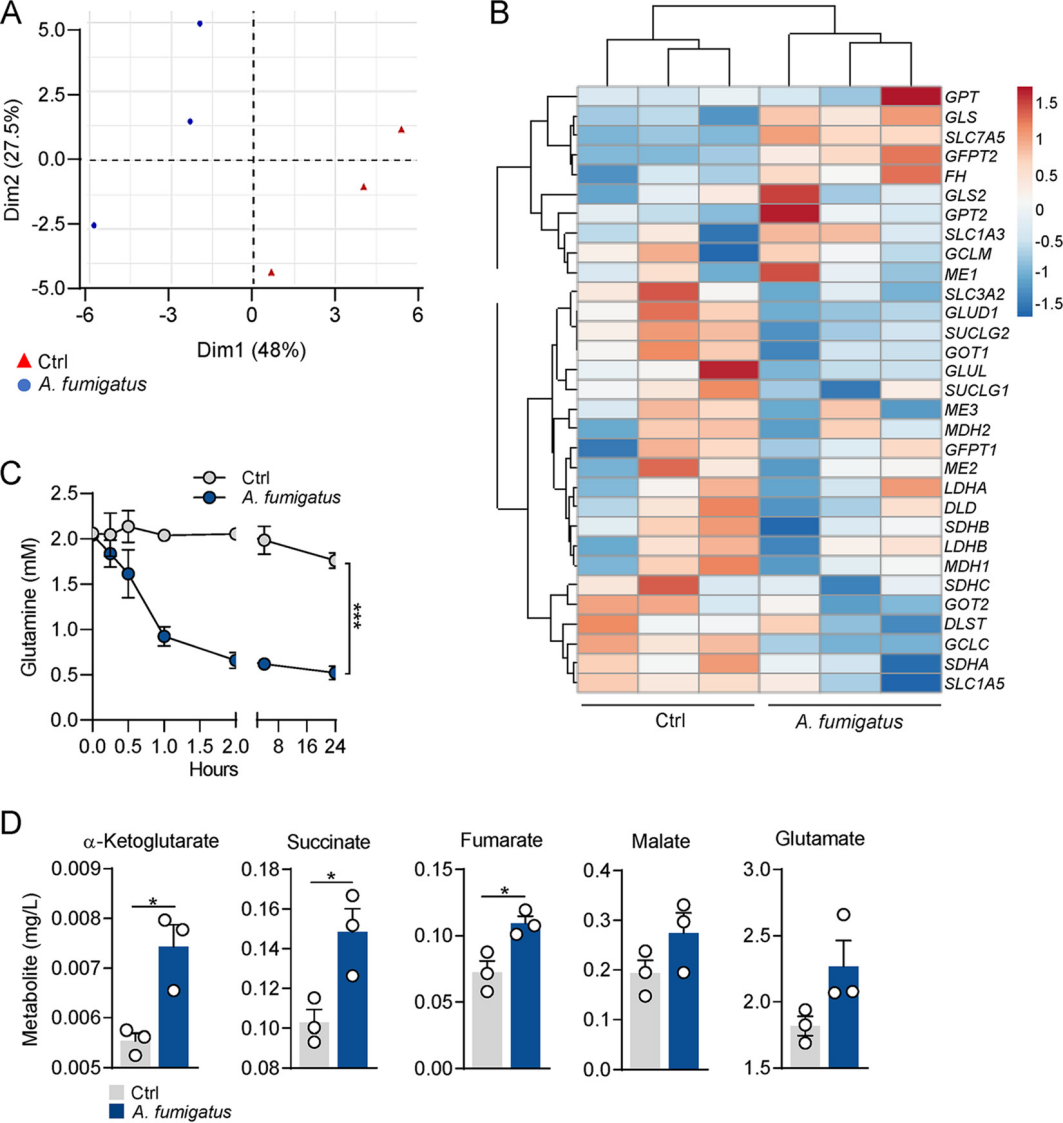
A. fumigatus induces glutamine metabolism in macrophages. (A) Principal-component analysis of the glutamine gene signatures of macrophages left noninfected (Ctrl) or infected with A. fumigatus, with the proportion of variance per principal component indicated between parentheses. (B) Heatmap of the expression pattern of glutamine genes in macrophages left noninfected (Ctrl) or infected with A. fumigatus for 2 h (*n* = 3). Expression of glutamine genes is presented as centered, by hierarchical clustering, and with scaled log_2_ fluorescence intensity (blue and red keys). (C) Glutamine consumption by macrophages left noninfected (Ctrl) or infected with A. fumigatus for 24 h (*n* = 3). (D) Levels of α-ketoglutarate, succinate, fumarate, malate, and glutamate (*n* = 3) in macrophages left noninfected (Ctrl) or infected with A. fumigatus for 6 h. Data are expressed as mean values ± SEM; *, *P* < 0.05, ***, *P* < 0.001 by two-way ANOVA with Tukey’s multiple-comparison test or Student’s two-tailed *t* test.

Our first aim was to investigate the modulation of glutamine metabolism in macrophages in response to A. fumigatus infection. Using available transcriptomic data from human macrophages infected with A. fumigatus (11), we started by evaluating whether the expression of genes involved in glutamine metabolism was modulated in response to infection. Principal-component analysis (PCA) of glutamine metabolism genes revealed that the expression profile of infected macrophages is different from noninfected controls (Fig. 1A). Likewise, unsupervised hierarchical clustering reveals that infected macrophages cluster separately from noninfected cells (Fig. 1B). Glutaminase (GLS), the amino acid transporter SLC7A5 (solute carrier family 7 member 5), and glutamine-fructose-6-phosphate transaminase 2 (GFPT2) were among the genes that were upregulated in the infected macrophages. The transcriptional induction of glutaminolysis was confirmed by the rapid glutamine consumption observed early after infection of macrophages relatively to noninfected cells (Fig. 1C). Given the contribution of glutamine metabolism to the replenishment of the TCA cycle, we next assessed the intracellular levels of several TCA intermediates using liquid chromatography tandem-mass spectrometry (LC-MS/MS) after infection of macrophages. Our results reveal a significant increase in TCA intermediates derived from glutamine metabolism, namely, α-ketoglutarate, succinate, and fumarate (Fig. 1D). Although they did not reach statistical significance, glutamate concentrations also tended to be higher in infected macrophages relative to noninfected controls.

### Antifungal effector functions of macrophages are decreased following glutamine depletion.

**FIG 2:**
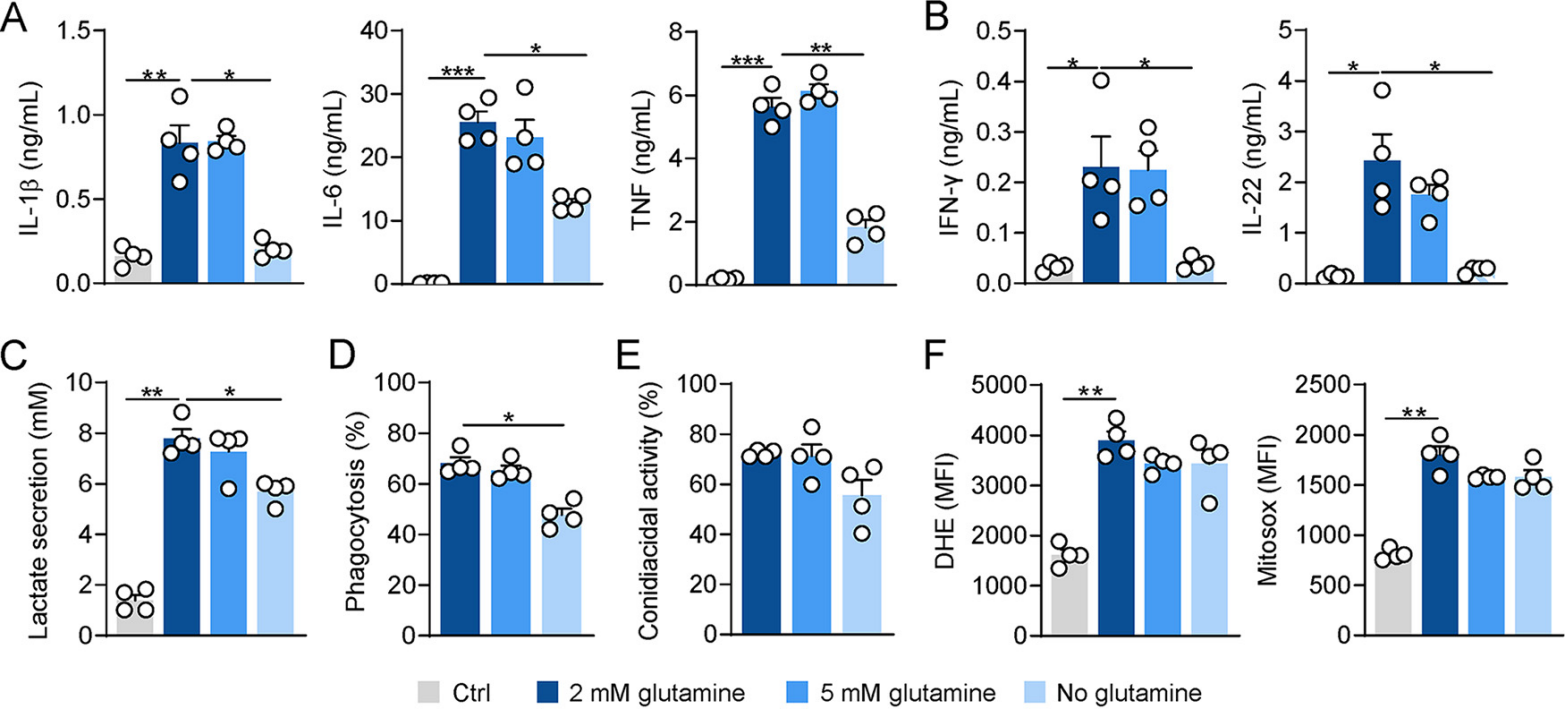
Glutamine depletion impairs antifungal effector functions of macrophages. (A) Levels of IL-1β, IL-6, and TNF secreted by macrophages left noninfected (Ctrl) or infected for 24 h with A. fumigatus in media without or with 2 mM or 5 mM glutamine (*n* = 4). (B) Levels of IFN-γ and IL-22 secreted by PBMCs left non-stimulated (Ctrl) or stimulated for 7 days with A. fumigatus in media without or with 2 mM or 5 mM glutamine (*n* = 4). (C) Lactate secretion by macrophages left noninfected (Ctrl) or infected for 24 h in media without or with 2 mM or 5 mM glutamine (*n* = 4). (D) Phagocytosis (*n* = 4) and (E) conidiacidal activity (*n* = 4) of macrophages in media without or with 2 mM or 5 mM glutamine. (E) Levels of cytosolic ROS (DHE, left) and mitochondrial ROS (Mitosox, right) by macrophages left noninfected (Ctrl) or infected for 4 h in media without or with 2 mM or 5 mM glutamine. Data are expressed as mean values ± SEM; *, *P* < 0.05; **, *P* < 0.01; ***, *P* < 0.001 by Student’s two-tailed *t* test.

The production of proinflammatory cytokines, phagocytosis, and fungal clearance are among the most relevant effector functions required for protective immune responses to A. fumigatus (22). We tested, therefore, how the depletion of glutamine could affect these processes during fungal infection. We confirmed that glutamine depletion resulted in a notable impairment in the production of proinflammatory cytokines such as IL-1β, IL-6, and TNF by macrophages (Fig. 2A), as well as the production of IFN-γ and IL-22 by PBMCs (Fig. 2B). To investigate whether the decreased cytokine production upon glutamine deprivation was associated with altered catabolism to lactate, we measured the concentrations of secreted lactate in culture supernatants. Our results show that lactate secretion following glutamine deprivation is impaired after infection compared to noninfected controls (Fig. 2C), suggesting that glutamine metabolism could also be fueling lactate production upon fungal infection.

Under glutamine-deprived conditions, macrophages also displayed an impaired phagocytic ability (Fig. 2D), a finding supported by data showing that phagocytosis is inhibited upon oxidative phosphorylation inhibition (13). In contrast, the conidiacidal activity was not compromised by depletion of glutamine (Fig. 2E), and, in line with this, no major alterations in ROS production were observed (Fig. 2F). Of note, glutamine supplementation failed to improve the overall antifungal effector capacity of macrophages (Fig. 2A to F).

### Inhibition of glutamine transport and metabolism impairs antifungal effector functions.

**FIG 3:**
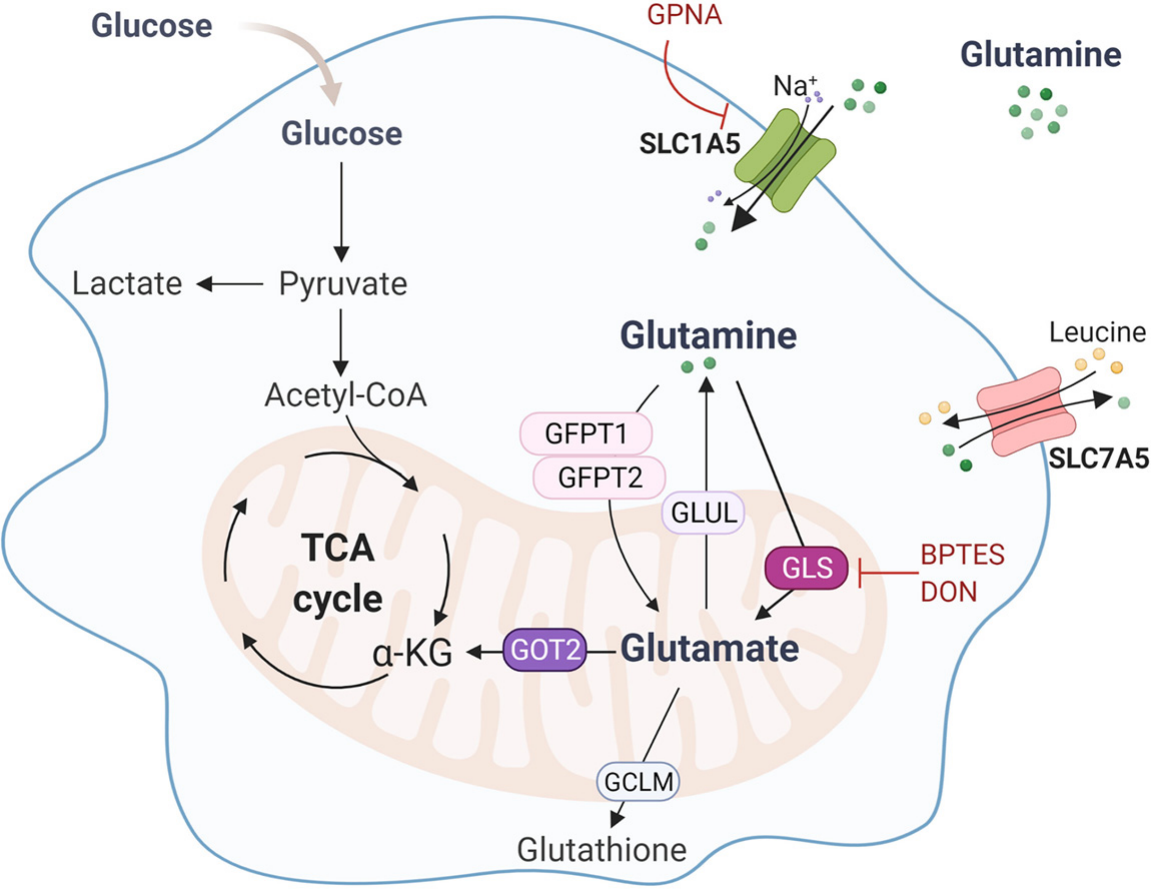
Schematic diagram of the glutamine metabolism pathways and the site of action of inhibitors.

**FIG 4:**
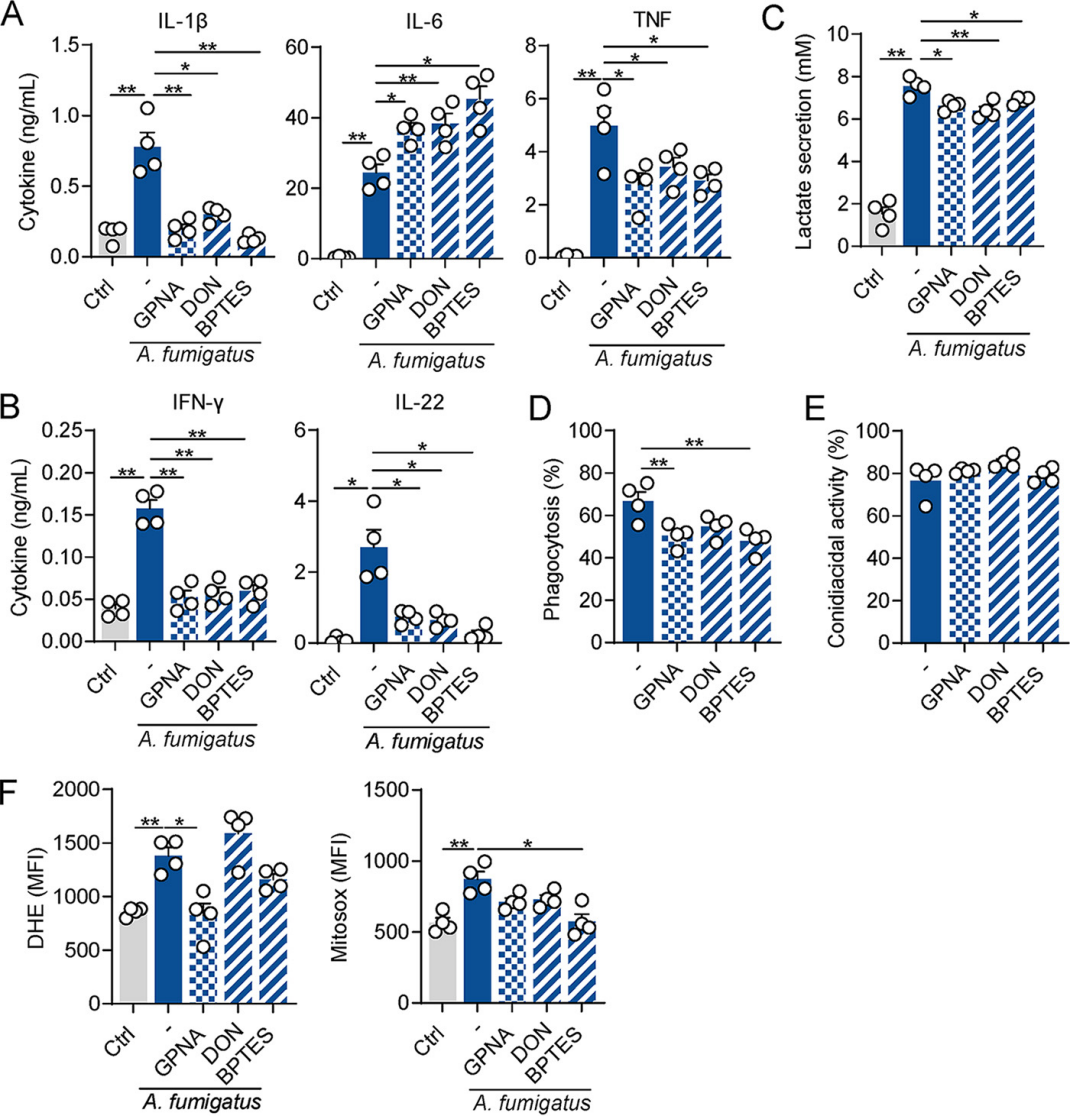
Pharmacological inhibition of glutamine metabolism at different steps impairs macrophage responses to A. fumigatus. (A) Levels of IL-1β, IL-6, and TNF secreted by macrophages infected for 24 h with A. fumigatus (*n* = 4), and (B) levels of IFN-γ and IL-22 secreted by PBMCs stimulated for 7 days with A. fumigatus (*n* = 4) and left untreated (-) or treated with GPNA, DON or BPTES. Uninfected cells were used as control (Ctrl). (C) Lactate secretion by macrophages infected for 24 h with A. fumigatus, and left untreated (-) or treated with GPNA, DON or BPTES (*n* = 4). Uninfected cells were used as control (Ctrl). (D) Phagocytosis and (E) conidiacidal activity of macrophages infected with A. fumigatus, left untreated (-) or treated with GPNA, DON or BPTES (*n* = 4). (F) Levels of cytosolic ROS (left) and mitochondrial ROS (right) in macrophages infected for 4 h with A. fumigatus and left untreated (-) or treated with GPNA, DON or BPTES (*n* = 4). Uninfected cells were used as control (Ctrl). Data are expressed as mean values ± SEM; *, *P* < 0.05; **, *P* < 0.01 by Student’s two-tailed *t* test.

To identify the steps in glutamine metabolism that were required for the effector mechanisms of macrophages, we resorted to specific pharmacologic inhibitors (Fig. 3). Treatment of macrophages with GPNA, a competitive inhibitor of glutamine uptake by the SLC1A5 transporter, decreased the production of IL-1β and TNF, but not IL-6, which was instead increased, in infected macrophages (Fig. 4A). The production of both IL-22 and IFN-γ was also compromised after inhibition of glutamine uptake in PBMCs (Fig. 4B).

Next, we assessed the effect of the inhibition of glutaminase, an enzyme that catalyzes the first step of glutaminolysis and converts glutamine into glutamate, using two different compounds: DON, a broad-spectrum glutaminase inhibitor, and BPTES, a specific inhibitor of mitochondrial glutaminase. These treatments promoted a similar defect in cytokine production in infected macrophages as described above, characterized by lower production of IL-1β and TNF, but not IL-6 secretion, whose levels were instead increased, similar to the GPNA treatment (Fig. 4A). The amounts of IL-22 and IFN-γ produced by PBMCs were also lower after the pharmacological inhibition of glutaminase activity (Fig. 4B).

Given that our results demonstrated a decreased lactate production in glutamine-depleted condition (Fig. 2C), we next sought to validate this finding using the inhibitors described above. Under these conditions, the production of lactate was compromised after the inhibition of either the transporter or glutaminase activity (Fig. 4C). The ability of macrophages to phagocytose the fungus was also impaired in all tested conditions (Fig. 4D), suggesting the need for both glutamine import and catabolism for the proper internalization of the fungus. Similar to what was observed in glutamine-deprived conditions, no differences were observed regarding the conidiacidal activity of macrophages treated with all inhibitors (Fig. 4E). Despite these observations, transporter inhibition in infected macrophages resulted in a decreased production of cellular ROS. In contrast, treatment with BPTES resulted in defective production of mitochondrial ROS (Fig. 4F), a finding in line with differential requirements for glutamine metabolism to ROS production.

### *In vivo* inhibition of glutamine metabolism increases susceptibility to experimental aspergillosis.

**FIG 5:**
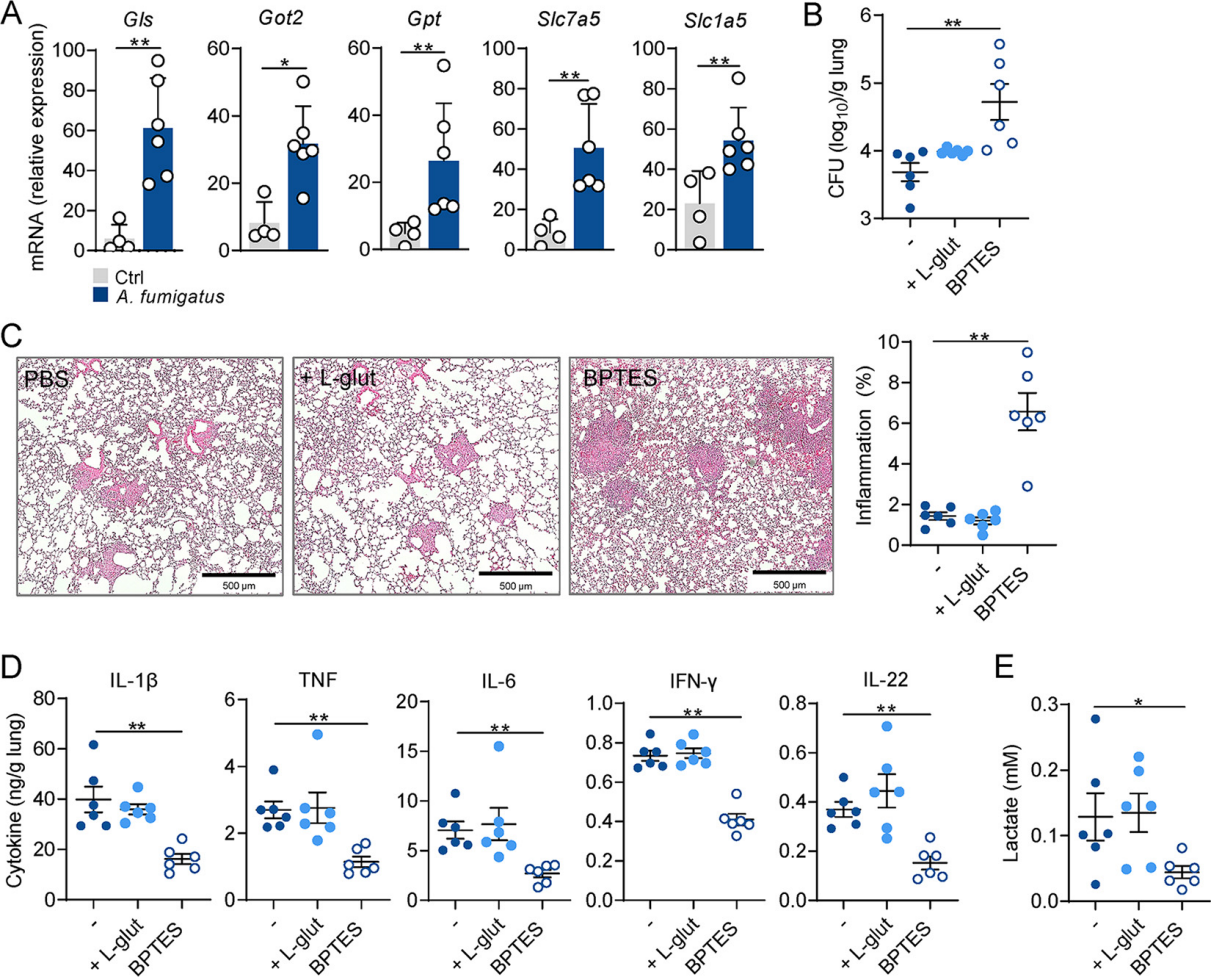
Glutamine synthase inhibition *in vivo* increases susceptibility to aspergillosis. (A) mRNA expression of *Gls*, *Got2*, *Gpt*, *Slc7a5*, and *Slc1a5* in the lungs of mice after 3 days of infection with A. fumigatus (*n* = 6). Naïve mice were used as control (Ctrl). (B) Fungal burden (log_10_) per gram of lung tissue was determined after 3 days of infection in mice treated with vehicle (PBS), l-glutamine supplementation or BPTES (*n* = 6, representative of three independent experiments). (C) Representative H&E-stained lung sections of infected mice treated with vehicle (PBS), l-glutamine supplementation or BPTES. The graph indicates the area of inflammation (%) within the whole tissue section. (D) Levels of IL-1β, IL-6, TNF, IFN-γ, and IL-22 (*n* = 6) and (E) lactate (*n* = 6) in lung homogenates of infected mice treated with vehicle (PBS), l-glutamine supplementation or BPTES. Data are expressed as mean values ± SEM; *, *P* < 0.05; **, *P* < 0.01 by Student’s two-tailed *t* test.

In light of the data demonstrating a pivotal role for macrophage glutamine metabolism for antifungal host defense, we next sought to validate this requirement in an *in vivo* model of pulmonary aspergillosis. Immunocompetent C57BL/6 mice were treated with BPTES or PBS prior to intranasal A. fumigatus infection. In support of the activation of glutamine metabolism in response to infection, we detected a significant induction of genes involved in glutamine import and utilization, such as *Gls* and *Slc1a5*, in the lungs of mice after A. fumigatus infection (Fig. 5A). Inhibition of glutamine metabolism rendered mice more susceptible to infection, as revealed by the increased fungal burden (Fig. 5B) and inflammatory pathology in the lungs (Fig. 5C). In accordance with the *in vitro* data, production of both proinflammatory and T-cell-mediated cytokines in lung homogenates of infected mice subjected to glutaminolysis inhibition were also lower than in noninfected controls under the same conditions (Fig. 5D). In line with these findings, the concentrations of lactate in the lungs of infected mice were also lower relatively to noninfected animals (Fig. 5E). On the other hand, glutamine supplementation failed to improve the overall ability of mice to deal with A. fumigatus infection (Fig. 5B to E): this argues that if cellular capacity to use glutamine is normal, additional supplementation does not further improve the outcome.

### Genetic variants in glutamine metabolism genes influence cytokine production.

We next examined whether genetic variation in the genes involved in the glutamine pathway could affect the host response to A. fumigatus. To do so, we tested the association of common single nucleotide polymorphism (SNPs) (minor allele frequency, ≥5%) in relevant candidate genes with differential cytokine production after fungal stimulation of PBMCs from healthy individuals of the 500FG cohort in the Human Functional Genomics Project (HFGP) (23). We identified a total of 12 cytokine quantitative trait loci (cQTLs) in genes involved in glutamine metabolism (Table 1). Several suggestive cQTLs with a nominal *P* value < 0.05 were identified, particularly in the gene encoding the glutamine transporter *SLC7A5*, with the rs11117336 SNP influencing the production of both IFN-γ and IL-22 by human PBMCs in response to A. fumigatus stimulation.

**TABLE 1:** Cytokine and expression quantitative trait loci of glutamine metabolism genes^*a*^

Gene	SNPs	cQTLs				eQTLs	Gene function
		IL-22	IFN-γ	TNF	IL-6		
GCLM	rs4598495	0.034				3.01E-08	Converts l-cysteine and l-glutamate to glutathione
GFPT1	rs62134901		0.010			3.88E-83	Converts l-glutamine to l-glutamate and d-fructose-6-phosphate to d-glucosamine-6-phosphate
	rs66693186			0.016		7.85E-232	
GFPT2	rs77017168			0.030		4.25E-16	
GLS2	rs7302925	0.037				1.47E-05	Converts glutamine to glutamate
GLUL	rs2924458	0.012				7.35E-13	Converts l-glutamate to l-glutamine
GOT2	rs1473206		0.024			4.40E-161	Converts l-aspartate to l-glutamate
	rs8058982	0.011				1.96E-41	
SLC1A5	rs314660				0.017	1.49E-05	High-affinity glutamine transporter
SLC7A5	rs11117336		6.80E-05			5.29E-14	Antiporter; exchanges intracellular glutamine for extracellular leucine
	rs11117336	1.71E-06				5.29E-14	
	rs4264398			0.018		2.59E-07	

a SNP, single-nucleotide polymorphism; QTLs, quantitative trait loci; IFN, interferon; IL, interleukin; TNF, tumor necrosis factor. *P* values for cytokine QTLs were obtained after correcting for age, gender, and cell counts. Only data with a nominal *P* value <0.05 are shown.

We next assessed the impact of the identified cQTLs on gene expression, using a publicly available meta-analysis of expression QTLs (eQTLs) in nontransformed peripheral blood samples available from the eQTLGen Consortium (24). Importantly, all of the identified cQTLs resulted being also known eQTLs (Table 1), therefore confirming that these variants can regulate both gene expression and cytokine production. Collectively, our data demonstrated that glutamine metabolism is an essential process required for the host defense against A. fumigatus infection.

## DISCUSSION

A growing body of evidence supports the importance of cellular metabolism during immune activation and host defense against fungal infections (11, 12, 21). It is nowadays well established that glucose metabolism in immune cells is essential for the deployment of antifungal effector functions, including the production of proinflammatory cytokines and other inflammatory mediators, as well as ROS (25). However, the intricate cross talk between the various metabolic pathways implies that pathways other than glycolysis may also play a role in the immune response to fungal infection. Herein, we examined the role of glutamine metabolism in the immune response to A. fumigatus infection. We found that glutamine-related genes were modulated at the transcriptional level during infection, and that glutamine depletion or pathway inhibition impaired the antifungal effector functions of macrophages. In support of these findings, SNPs in genes involved in glutamine metabolism were found to influence cytokine production in response to A. fumigatus stimulation. Collectively, these data highlight the central role of glutamine metabolism of immune cells in aspergillosis.

In immune cells, glutamine metabolism is required for amino acid and nucleotide synthesis, NADPH and energy production, and several other biosynthetic pathways involved in cell proliferation and function (26). Our results indicate that glutamine metabolism inhibition affects both monocyte-derived (TNF, IL-6, and IL-1β) and T-cell-derived (IFN-γ and IL-22) cytokine production. While a rapid glutamine import and metabolism is essential to the activation and differentiation of naive T-cells, leading to the induction of T helper 1 (Th1) and Th17 cells, and enhanced inflammatory T cell responses in mouse models of immunity and autoimmunity (27), our results also support a relevant role for glutaminolysis in macrophage function. In these cells, glutamine is also utilized at high rates and contributes to the definition of functional phenotypes depending on the metabolic routes used (28). Glutamine typically favors an antiinflammatory phenotype of macrophages through the generation of α-ketoglutarate, which is essential for oxidative phosphorylation and fatty acid oxidation, and contributes to epigenetic modifications such as the demethylation of H3K27 on the promoters of M2-specific marker genes (29). In this regard, glutaminolysis has been reported to be required for the epigenetic modifications required for the induction of innate immune memory in response to fungal β-1,3-glucan stimulation (15).

In certain conditions, however, glutamine utilization through the Krebs cycle can also favor the generation of succinate to promote the polarization of inflammatory M1-like macrophages (30). In our experimental model, macrophages subjected to fungal stimulation displayed an accumulation of intracellular succinate, which may indicate that glutamine is utilized to favor inflammatory programs required to effectively counteract fungal infection. This result is in line with the data showing that macrophages infected with M. tuberculosis and patients with active tuberculosis also display an increased transcription of genes related to the glutamine pathway (19). Moreover, lactate secretion is also decreased under conditions of impaired glutaminolysis, which suggests an important cross talk between these two metabolic routes pathways and that may contribute to the overall observed phenotype.

Besides cytokine production, inhibition of glutamine metabolism was also found to impair phagocytosis but, remarkably, not fungicidal activity. In this regard, low glutamine concentrations were associated with reduced phagocytosis by murine peritoneal macrophages (31). In contrast, oral glutamine supplementation increased the activity of immune cells such as neutrophils, including phagocytosis, in experimental sepsis (32). Moreover, glutamine supplementation also fostered phagocytosis by neutrophils and monocytes from postoperative patients *in vitro* (33). Although the mechanistic link between glutamine metabolism and phagocytosis remains elusive, it may imply changes to the cytoskeletal organization. In this regard, glutamine utilization through a noncanonical pathway independent of the traditional requirement of glutamate dehydrogenase to fuel the generation of ɑ-ketoglutarate was reported to foster the cellular requirements of efferocytosis and high-energy cytoskeletal rearrangements (34). The apparently relevant role for glutamine metabolism on phagocytosis is in line with the rapid consumption of glutamine by infected macrophages, which seems to be required for an early response to the fungus. Instead, glycolysis appears to be more relevant to mechanisms of fungal elimination, including ROS production, a possibility that is also supported by the delayed kinetics of glucose consumption and lactate secretion after infection compared to glutamine (11).

In our *in vitro* and *in vivo* models, glutamine supplementation failed to improve the outcome of the fungal infection. This may be related to a threshold effect, above which glutamine supplementation does not add to the efficiency of the metabolism route for its utilization. A recent study has shown that while glutamine is essential for the control of Leishmania donovani infection, its supplementation failed to improve the outcome of the infection (20). Interestingly, glutamine instead potentiated parasite clearance during miltefosine treatment, suggesting that glutamine might also favor the therapeutic efficacy of antifungal therapies and act as a promising adjuvant in the treatment of fungal infections.

The clinical course of fungal infection is highly variable, and studies in patients with rare mutations and cohort-based studies suggest that the trajectory of disease is largely defined by the genetic background of the patient (35, 36). Our results demonstrating that SNPs in glutamine pathway genes control cytokine production in response to fungal stimulation provide further support to the role of glutamine metabolism in the host defense against A. fumigatus. In this regard, genetic variation in 6-phosphofructo-2-kinase/fructose-2,6-biphosphatase 3 (PFKFB3), a critical regulator of glucose metabolism, was found to impair antifungal effector functions of macrophages and predispose recipients of allogeneic hematopoietic stem-cell transplantation to the development of invasive pulmonary aspergillosis (37). These observations suggest that the interindividual genetic regulation of glutamine metabolism genes may also define divergent immune phenotypes and profiles of susceptibility to fungal infection and highlight the possibility that genetic and immunometabolic signatures may represent clinically important tools to identify and stratify patients.

Our study has also limitations to be acknowledged. The functional parameters we have studied represent only a few of the many components of the host immune response against A. fumigatus. The remarkable susceptibility of mice to experimental aspergillosis following the inhibition of glutaminolysis supports the likely involvement of glutamine metabolism in multiple effector mechanisms affecting immune (and nonimmune) cells other than monocytes/macrophages and T-cells. Also, the association of SNPs in glutamine genes with the development of fungal infection in patients at-risk, as well as their detailed characterization besides the impact on cytokine production, is yet to be fully explored.

In conclusion, our study has identified glutamine metabolism as a fundamental metabolic process needed to mount an effective host response against A. fumigatus. In previous studies, we have identified other metabolic pathways, namely, glycolysis, to be relevant for host responses in aspergillosis (11, 37). These studies and others underline the relevance of immune cell metabolism for the response to fungal infection and its potential role in developing new preventive or therapeutic strategies for aspergillosis (38).

## MATERIALS AND METHODS

### Ethics statement.

The functional experiments involving cells isolated from the peripheral blood of healthy volunteers at the Hospital of Braga, Portugal, were approved by the Ethics Subcommittee for Life and Health Sciences (SECVS) of the University of Minho, Portugal (no. 014/015). The Human Functional Genomics Project (HFGP) study was approved by the Ethical Committee of Radboud University Nijmegen, the Netherlands (no. 42561.091.12). Experiments were conducted according to the principles expressed in the Declaration of Helsinki, and participants provided written informed consent.

### Mice.

C57BL/6 mice purchased from Charles River Laboratories were bred under specific pathogen-free conditions and kept at the animal facilities at the Life and Health Sciences Research Institute (ICVS). Mice were fed *ad libitum* and maintained under light/dark cycles of 12 h, temperature of 18–25°C, and humidity 40–60%. Animal experimentation was performed on eight to twelve-week-old gender-matched mice, following biosafety level 2 (BSL-2) protocols approved by the Institutional Animal Care and Use Committee (IACUC) of the University of Minho, and both ethical and regulatory approvals were consented by CEICVS (no.074/016). All procedures followed the EU-adopted regulations (Directive 2010/63/EU) and were conducted according to the guidelines of Direção-Geral de Alimentação e Veterinária (DGAV).

### Aspergillus strains and culture conditions.

A. fumigatus was grown on 2% malt extract agar for 7 days at 28°C. The conidia were harvested from agar plates using phosphate buffer saline (PBS) (Gibco) with 0.05% Tween 20 (Sigma-Aldrich), followed by gentle agitation and subsequent filtration through a 40-μm-pore size cell strainer (Falcon). The concentration of conidia/mL was determined by counting in a Neubauer chamber.

### Isolation of PBMCs and generation of macrophages.

Peripheral blood mononuclear cells (PBMCs) were enriched from buffy coats from healthy donors by density gradient using Histopaque-1077 (Sigma-Aldrich), washed twice with PBS (Gibco), and resuspended in RPMI 1640 culture medium with 2 mM glutamine (Thermo Fisher) supplemented with 10% human serum (Sigma-Aldrich), 10 U/mL penicillin/streptomycin and 10 mM HEPES (Thermo Fisher). Monocytes were isolated from PBMCs by resorting to a positive magnetic bead separation with anti-CD14^+^-coated beads (Miltenyi) according to the manufacturer’s instructions. To evaluate the purity of the isolated monocytes, 5 × 10^5^ cells were stained with BV 650 anti-human CD14 (clone M5E2) antibody for 30 min at 4°C. Cell viability was assessed by staining with Zombie Green fluorescent dye (BioLegend) for 30 min at 4°C. Pellets were washed and resuspended in FACS buffer (PBS containing 2% FBS and 2 mM EDTA) prior to analysis. Data were obtained on a BD FACS LSRII instrument (Becton, Dickinson) and processed using FlowJo (Tree Star Inc.). The obtained CD14^+^ population displayed a purity higher than 94%, and with more than 97% viable cells. Isolated monocytes were then resuspended in a complete RPMI medium and seeded at a concentration of 1 × 10^6^ cells/mL in 24-well or 96-well plates (Corning) and left for 7 days with 20 ng/mL of recombinant human granulocyte-macrophage colony-stimulating factor (GM-CSF, Miltenyi) at 37°C and 5% CO_2_. The culture medium was replaced every 3 days, and acquisition of macrophage morphology was confirmed by visualization in a BX61 microscope (Olympus).

### Cell stimulations and treatments.

Unless otherwise indicated, cells (5× 10^5^/well in 24-well plates) were left noninfected or infected with live conidia of A. fumigatus at a 1:10 effector-to-target ratio for 24 h at 37°C and 5% CO_2_. The experiments involving glutamine depletion or supplementation used RPMI 1640 medium without glutamine and glutamine (Thermo Fisher). To interfere with glutamine metabolism, macrophages were pretreated for 1 h with 600 μM L-γ-Glutamyl-p-nitroanilide (GPNA), 10 μM 6-Diazo-5-oxo-l-norleucine (DON), and 50 μM Bis-2-(5-phenylacetamido-1,3,4-thiadiazol-2-yl)ethyl sulfide (BPTES) (all from Sigma-Aldrich). Viability and expression of surface markers (HLA-DR, CD86, CD163, and CD206) were evaluated in all experimental conditions to test for population homogeneity. Macrophages were viable under all conditions tested. Data were assessed in triplicates or quadruplicates and are shown as the mean value for each individual.

### RNA sequencing.

RNA sequencing was performed as previously described (11). Briefly, macrophages (5 × 10^5^ cells/mL in 24-well plate) were left noninfected or infected with live conidia of A. fumigatus at a 1:2 effector-to-target ratio for 2 h. Samples were processed, sequencing, and analyzed at IMGM Laboratories GmbH (Germany). Data have been deposited in Gene Expression Omnibus (GEO) with the accession code GSE128661. To better visualize the most significantly represented genes of a specific functional class, heatmaps were created using ClustVis: a web tool for visualizing the clustering of multivariate data (http://biit.cs.ut.ee/clustvis/). PCA for this data set was conducted using R software v4.2.0.

### Quantification of glutamine.

For the quantification of glutamine, macrophages (5 × 10^5^/well in 24-well plates) were infected for 15 min, 30 min, 1h, 2h, 6h, and 24h at 37°C for 5% CO_2_ with live A. fumigatus conidia at a 1:10 effector-to-target ratio. After infection, supernatants were collected and centrifuged, and glutamine levels were quantified using the Glutamine and Glutamate Determination kit (Sigma-Aldrich), following the manufacturer’s instructions.

### LC-MS/MS-targeted metabolomics.

Macrophages (5 × 10^5^ cells/mL in 24-well plate) were infected with A. fumigatus conidia at a 1:2 effector-to-target ratio for 6 h at 37°C in 5% CO_2_. Cells were detached with accutase (GRiSP), and the resulting cell suspensions were pooled from three different wells. The resulting cell suspensions were centrifuged at 4°C, the supernatant discarded, and the resulting pellet was immediately frozen in liquid nitrogen to quickly quench the cell metabolism. The analysis of the metabolites was performed with liquid chromatography-mass spectrometry (LC-MS/MS). The analysis was performed in an Agilent 1290 Infinity using a 1290 Infinity Binary Pump (1200 bar) and a 1260 Infinity Quaternary Pump (400 bar). The LC system was coupled to an Agilent 6470 triple-quadrupole mass spectrometer using an electrospray ionization (ESI) interface working in multiple reaction monitoring (MRM) mode. The chromatographic method used HILIC interaction chromatography (Agilent InfinityLab Poroshell). MRM transitions were chosen according to the Metabolomics dMRM library and method from Agilent Technologies (p/n G6412AA).

### Mouse infection.

For the *in vivo* studies, from day 0 (day of infection) and until the end of the experimental protocol, 500 mg/kg of glutamine and 150 μg/Kg BPTES were administered to mice daily via oral gavage or via the intraperitoneal route (i.p.), respectively. Control groups consisted of mice to which an equal volume of vehicle (sterile PBS) was administered. On day 0, mice were challenged with 1 × 10^8^ live conidia of A. fumigatus (A1163 strain) by a noninvasive intranasal (i.n.) infection procedure upon anesthesia with 5 mg/Kg of ketamine (Ketamidor, Ritcher Pharma) and 1 mg/Kg of medetomidine (Domtor, Ecuphar), and weight and well-being were monitored during the infection period. At 3-days postinfection, mice were sacrificed, and the lungs were PBS-perfused and excised. To assess the fungal burden, the right lobes were removed, weighted, and homogenized in 1 mL of sterile PBS with a tissue homogenizer (Glas-Col). The resulting homogenate was serially diluted and plated on solid growth media. The resulting CFU were counted, and the fungal burden was calculated log_10_ CFU/g of lung.

### Inflammation analysis.

For the histological analysis, at 3-days postinfection, lungs were perfused with PBS, excised, and fixed in buffered formalin solution 10% (Sigma-Aldrich) for 48 h under agitation at room temperature. Lungs were then processed, paraffin-embedded, and lung sections of 5 μm thickness were stained with hematoxylin and eosin (H&E) to assess inflammation. The images were acquired on a BX61 widefield upright microscope using a DP70 high-resolution camera (Olympus), and the area of inflammation was determined using ImageJ software v1.50i (Fiji).

### Phagocytosis assay.

To evaluate phagocytosis, macrophages (5 × 10^5^/well in 24-well plates) were infected with fluorescein isothiocyanate (FITC)-labeled conidia of A. fumigatus at a 1:5 effector-to-target ratio. The incubation period was synchronized for 30 min at 4°C, and phagocytosis was initiated by shifting the coincubation to 37°C and 5% CO_2_ for 1 h. Phagocytosis was stopped by washing wells with ice-cold PBS, and extracellular conidia were stained with 0.25 mg/mL Calcofluor White (Sigma-Aldrich) for 15 min at 4°C to avoid further ingestion. The cells were detached with accutase and resuspended in PBS. The percentage of macrophages with ingested green conidia was collected on a BD FACS LSRII instrument (Becton, Dickinson) and processed using FlowJo v10.5.3 (Tree Star Inc.). Unstained samples were used to set voltages.

### Conidiacidal activity assay.

Macrophages (1 × 10^5^/well in 96-well plates) were infected with A. fumigatus conidia at a 10:1 effector-to-target ratio. To allow the internalization of conidia, cells were incubated for 1 h at 37°C and 5% CO_2_. Medium containing the noningested conidia was removed, and cells were washed twice with prewarmed PBS. To measure the conidiacidal activity, macrophages were allowed to eliminate the internalized conidia for 2 h at 37°C in 5% CO_2_. After incubation, culture plates were snap-frozen at −80°C and thawed at 37°C to cause the cell lysis and release of internalized conidia. Serial dilutions of cell lysates were plated on solid growth media and incubated at 37°C for 24 h. The number of CFU was counted, and the percentage of eliminated conidia was calculated.

### Measurement of ROS production.

Macrophages (5 × 10^5^/well in 24-well plates) were infected for 4 h at 37°C in 5% CO_2_ with live A. fumigatus conidia at a 1:5 effector-to-target ratio. The supernatant was removed, and cells were detached with accutase. The cell suspensions were collected, centrifuged for 5 min at 2,000 rpm, and then 10 μM dihydroethidium (DHE; Thermo Fisher) or 5 μM Mitosox (Thermo Fisher) were added, before incubation for 30 min at 37°C, protected from light. Cytosolic and mitochondrial reactive oxygen species (ROS) were measured on a BD FACS LSRII instrument (Becton, Dickinson) and processed using FlowJo v10.5.3 (Tree Star Inc.).

### Cytokine measurements.

Macrophages (5 × 10^5^/well in 24-well plates) were infected with A. fumigatus live conidia at a 1:10 effector-to-target for 24 h, while PBMCs (2.5 × 10^6^/well in 96-well plates) were infected with A. fumigatus heat-killed conidia at a 1:4 effector-to-target ratio for 7 days at 37°C and 5% CO_2._ Then, the supernatants were collected, and cytokine levels were quantified using ELISA MAX Deluxe set Kits (BioLegend), according to the manufacturer’s instructions. Cytokine quantification was also performed on the supernatants of lung single-cell suspensions 3-day postinfection using ELISA MAX Deluxe set Kits (BioLegend).

### Quantification of lactate.

Macrophages (5 × 10^5^/well in 24-well plates) infected for 24 h at 37°C in 5% CO_2_ with live A. fumigatus conidia at a 1:10 effector-to-target ratio. Then the supernatants were removed and centrifuged, and lactate levels were determined using the enzymatic colorimetric lactate assay (SpinReact), following the manufacturer’s instructions. For the quantification of lactate *in vivo*, lungs were removed and homogenized in lactate assay buffer, and lactate levels were determined using the l-Lactate assay kit (Abcam), following the manufacturer’s instructions.

### RNA isolation and qRT-PCR.

The total RNA from the lungs was extracted using the GRS Total RNA kit-Tissue (GRiSP), on day 3 postinfection, according to the manufacturer’s instructions. The concentration and quality of total RNA in each sample were determined by spectrophotometry using the ND-100 UV-visible light spectrophotometer (NanoDrop). One microgram of the total RNA was retro-transcribed using the first-strand cDNA Synthesis kit (Nzytech). PowerUp SYBR green Master Mix (Applied Biosystems, Thermo Fisher Scientific) was used to perform quantitative PCR in an Applied Biosystems 7500 fast qPCR system (Applied Biosystems). Data were analyzed using 7500 Software v2.0.6 software (Applied Biosystems). Amplification efficiencies were validated, and the expression levels of the transcripts were normalized using the ubiquitin (*Ubb*) gene.

### Cytokine QTL mapping.

PBMC collection, stimulation experiments with A. fumigatus, and cQTL mapping was previously described (23). Briefly, genotype, cytokine, and cell count data measured by FACS for total lymphocytes data were available for 442 individuals from a population-based cohort, 500FG cohort, which is part of the HFGP (www.humanfunctionalgenomics.org). Log-transformed cytokine levels mapped to genotype data using a linear model with age, sex and cell counts as covariates. Given that the total number of independent tests among all phenotypes was much less than the number of phenotypes measured, correction for multiple testing was based on the number of SNPs tested for each trait, and a *P* value of < 5 × 10^−8^ was defined as the threshold for genome-wide significant cytokine QTLs. A nominal *P* value of < 0.05 was defined as the threshold for suggestive cQTLs.

### Statistical analysis.

The data were expressed as means ± SEM. Statistical significance of differences was determined by two-tailed Student's *t* test or two-way ANOVA with *post hoc* tests for multiple comparisons (*P < *0.05 was considered statistically significant). Analyses were performed in GraphPad Prism software v9.3.1.
